# Othering Older People’s Housing: Gaming Ageing to Support Future-Planning

**DOI:** 10.3390/ijerph21030304

**Published:** 2024-03-05

**Authors:** Vikki McCall, Alasdair C. Rutherford, Alison Bowes, Sadhana Jagannath, Mary Njoki, Martin Quirke, Catherine M. Pemble, Melanie Lovatt, Lisa Davison, Katie Maginn, Pat Scrutton, Ro Pengelly, Joan Gibson

**Affiliations:** Faculty of Social Sciences, University of Stirling, Stirling FK9 4LA, UK; alasdair.rutherford@stir.ac.uk (A.C.R.); a.m.bowes@stir.ac.uk (A.B.); sadhana.jagannath@stir.ac.uk (S.J.); mary.njoki1@stir.ac.uk (M.N.); martin.quirke@stir.ac.uk (M.Q.); catherine.pemble@stir.ac.uk (C.M.P.); melanie.lovatt1@stir.ac.uk (M.L.); lisa.davison@stir.ac.uk (L.D.);

**Keywords:** ageing in place, inclusive design, serious games, healthy ageing, co-production

## Abstract

The ‘othering’ of ageing is linked to an integrated process of ageism and hinders planning for the future for both individuals and practitioners delivering housing and health services. This paper aims to explore how creative interventions can help personalise, exchange knowledge and lead to system changes that tackle the ‘othering’ of ageing. The Designing Homes for Healthy Cognitive Ageing (DesHCA) project offers new and creative insights through an innovative methodology utilising ‘serious games’ with a co-produced tool called ‘Our House’ that provides insights into how to deliver housing for older people for ageing well in place. In a series of playtests with over 128 people throughout the UK, the findings show that serious games allow interaction, integration and understanding of how ageing affects people professionally and personally. The empirical evidence highlights that the game mechanisms allowed for a more in-depth and nuanced consideration of ageing in a safe and creative environment. These interactions and discussions enable individuals to personalise and project insights to combat the ‘othering’ of ageing. However, the solutions are restrained as overcoming the consequences of ageism is a societal challenge with multilayered solutions. The paper concludes that serious gaming encourages people to think differently about the concept of healthy ageing—both physically and cognitively—with the consideration of scalable and creative solutions to prepare for ageing in place.

## 1. Introduction

Planning for older age is a challenge and can be linked to an ‘othering’ of ageing. Othering occurs when people perceive differences alongside negative comparisons between people, groups, homes and wider environments. This encourages an understanding of ageing that is external to the individual, or their idea of themselves. In other words, people often view age as something outside their personal experience and use imaginary interpretations of what ageing is. This is reinforced by the othering of sub-groups of older people, for example the ‘older old’ (completely other) and ‘young-old’ (more like me) [1]. The othering of ageing encourages the perception that ageing simply happens to other people, making it a challenging process to personalise and is linked to dangerous, negative, and derogatory forms of ageism [2]. Ageism attached to ‘othering’ is a key barrier that stops individuals and professionals planning for the future. The Designing Homes for Healthy Cognitive Ageing (DesHCA) project has developed and tested tools to tackle the challenges wrapped up in the ‘othering’ of ageing via a focus on designs related to homes and communities. This paper presents evidence for innovative ways of tackling ageism and ‘othering’ through serious games that encourage positive interventions that support healthy ageing for individuals and professionals.

Developing interventions to support healthy ageing is important because when growing older is perceived to happen to other people, it can result in a lack of action to future-proof homes and environments to be ready for growing older and support diverse health needs. People tend to connect to ageing by focusing on ‘other’ groups, or types of housing, rather than changes and good design for themselves. Furthermore, the othering of ageing sits alongside social and housing policy that frames planning for housing and ageing as a ‘personal issue’ rather than a ‘public issue’ [3]. When adaptations to support ageing and diverse health needs are later required in homes, the process is seen as a ‘fight’ by both service users and those delivering such services [4]. Consequently, planning for housing and ageing is not taken as a priority on an individual, sectoral, or structural level.

The results of not planning the right homes and wider environments for ageing include high levels of unmet health and care needs for older people, impacted by the environment people live in [5]. Ageing is best supported by environments when they can adapt with people’s changing experiences [6]. Cognitively sustainable housing takes into consideration both cognitive and physical impairment. The impact of non-decent homes is not felt equally by all older people, with clear individual and spatial inequalities [7]. This is clear when looking at diverse experiences of ‘ageing in place’ and the important link between the right environment and the people it supports [8,9]. Planning for ageing to develop supportive, accessible, flexible homes and environments are important for independent and safe ageing in place. It allows people to project themselves into the future [10] and proactively plan for later life, making it an essential part of an effective preventative agenda. Yet, the othering of ageing discourages planning for ageing and the proactive consideration of home design features that could help sustain healthy ageing in place.

There is increasing interest in ’play’ as a strategy, research approach, and method to engage and motivate people to think about ageing differently [11,12,13]. The approach of utilising ‘serious games’, which employs gaming-type methods both online and in person, is designed to encourage, motivate, and change perspectives on serious topics such as ageing. This has been applied effectively in health promotion with older adults [13] and in helping policy makers, older people, and service providers plan for housing the ageing population [12].

DesHCA aims to test, understand, and identify facilitators and barriers for various stakeholders, including older people, in achieving cognitively sustainable housing, in both new-build and retrofit contexts. Planning successful housing for older age is clearly a challenge, and this article explores the following research questions: 1. Do housing professionals and wider stakeholders engage in the ‘othering’ of ageing? 2. How is ‘othering’ of older people reflected in housing, health, and social care practice? 3. Can creative methodologies such as serious gaming influence the ‘othering’ of ageing on a professional and personal level? 4. Can ’serious play’ support an increased understanding of healthy cognitive ageing and ageing in place? Exploring these questions helps give insight into how to overcome the problem of ‘othering’ of ageing, ageism and lack of future planning in housing and health services.

To overcome the ‘othering of ageing’, the DesHCA project has developed a co-produced tool called ‘Our House’ which is a serious game that supports insightful ways of delivering housing for older people that is cognitively sustainable and inclusive. Serious play, we argue, encourages people to think differently about the concept of healthy ageing—both physically and cognitively. This then allows us to consider scalable and creative solutions and prepare homes and environments for ageing in place.

### 1.1. The Othering of Ageing

Gerontologists have long accepted (since Butler 1969 [14]) that ageism is embedded in modern society, and that this has negative consequences for older people who may as a result be excluded from activities and assumed to be vulnerable or to lack competency. Since Butler, the concept has been widely used by researchers, sometimes uncritically [15]. Following an extensive review of definitions of ageism, Iverson et al. argued for clear definition of the concept, noting that it indicates prejudice or stereotyping of older people that can be explicit or implicit and can be expressed at different levels, including by the individual themselves [16]. Naughton et al.’s more recent overview illustrates that ageism can be conceptualised as a process of othering [17], with the older population categorised (following van Dyke 2016 [1]) as ‘abjected’ or ‘glorified’. For example, Gilleard, in exploring de Beauvoir’s work on the ‘other’ noted that people are “always seeming to age in ways other than one’s self” [18] (p. 286). ‘Abjection’ is used to other ‘fourth agers’—i.e., those experiencing physical and mental limitations, notably dementia (see also Gilleard and Higgs [19]). ‘Glorification’ is used to ‘other’ older people who are engaged in ‘active ageing’, demonstrating achievements that are considered remarkable in relation to expectations (i.e., stereotypes) of their age. Whilst specific modalities of othering are culturally specific, the process nevertheless has international consequences [20].

Recent renewed interest in the effects of ageism has been stimulated by the COVID-19 pandemic. Verbruggen et al. describe the pandemic as having functioned as a ‘magnifying glass’ for ageism [21]. Naughton et al. argue that societal responses to the pandemic demonstrated the pervasiveness of othering older people [17], and that the segregation and isolation of some older people in care facilities with its surrounding discourse of frailty and vulnerability has served to exacerbate othering of older people generally. People living with dementia and or physical limitations have been most notably affected [22]. The research drawn upon in this paper was conducted in an immediately post-pandemic context, in which the othering of older people had arguably been sharpened. It provides empirical evidence on the consequences of othering as well as a potential means of addressing this problematic process.

The consequences of ageism for older people are generally well documented internationally [23] and nationally in the UK [24] in terms of stereotyping, restrictions of access to resources, and poorer health and well-being. Researchers have frequently considered the impacts of ageism in employment and health well-being [25,26,27]. However, there is a dearth of research considering ageism in housing. Housing researchers have recently emphasised the importance of ‘ageing in the right place’ [9] in housing that can support us as we age and may experience physical, sensory and cognitive challenges [28], but there is a need for further consideration of how ageism comes into play in housing provision. In our exploration of this issue in the paper, two sets of consequences of ageism are particularly pertinent, including processes of self-ageism/self-othering, and thinking about the future in older age, as we focus on how othering by ageism operates as people make housing decisions and look towards both their future selves and the futures of others.

Self-ageism or self-othering in this context relates to a denial of one’s own ageing: the individual considers that other people age, those ‘others’ are old people, whereas I am ageless and feel the same as I have always done. It can also manifest as a feeling of not deserving support as older age presents challenges, because the process is inevitable and negative and as one is increasingly excluded from society, one feels worthy of exclusion. As such, self-ageism can have negative consequences for the individual. ‘Third agers’ who speak about older people as ‘other’ disassociate themselves from fourth age problems, like needing support as life becomes difficult [21]. In Verbruggen et al.’s view, claiming agelessness for oneself is a form of ageism with consequences for others as it will affect one’s actions towards them [21]. Schuurman et al. call for more research into self-ageism to demonstrate how it plays out in different cultural contexts [20]; our study is focused on ageism in housing decision making, including self-ageism.

Decision making is an inherently future-oriented activity; it requires consideration both of how actions in the present may affect future situations, and how anticipated or potential futures can be affected by present decisions. Imagining oneself in the future or conceptualising oneself as belonging in the future is, therefore, important to one’s agency. Deeply embedded ageism, however, which associates future ageing with decline [29] offers few incentives to actively consider and plan for one’s own future ageing self. This relates to the concept of ‘narrative foreclosure’ whereby people may feel that after a certain age the future has nothing more to offer them [30]. Empirical research has found that older participants have been reluctant to discuss their futures, instead reorienting discussions to their past achievements [31] or to future aspirations for family members [32].

The research literature includes calls to action (e.g., Grenier and Phillipson [22]), whilst also emphasising the difficulty of tackling the consequences of such deeply entrenched ideas (e.g., Naughton et al. [17]). Few researchers have explored empirically and in practical terms how the consequences of ageism might be addressed.

The paper focuses on a tool which exposes othering and challenges participants with the issues of othering older age, particularly the ‘abjection’ images that attach to people living with physical and/or cognitive impairment. The tool can contribute to countering the othering of ageing, both for ourselves as we age and for those charged with developing and providing appropriate housing in the context of an ageing population; and therefore support us as we age to live the lives we wish in the places where we prefer to do so.

### 1.2. Play and Serious Gaming to Personalise Ageing

Landers et al. [33] draw a distinction between ‘gamification’ (‘the implementation of game-like attributes outside of the context of a game’) and ‘serious games’, where games are used with education as their primary purpose, rather than entertainment. While serious gaming and game-based learning have been used in some fields, particularly economics, since the 1960s [34], it is only recently that these methods have begun gaining increased traction in academic, professional, and personal settings [35,36], representing an emerging research field [11].

Agogué et al. argue that game-based learning methods are highly successful at ideation, improving education outcomes, developing knowledge, and encouraging creativity [34]. Furthermore, Jacob et al. demonstrated the effectiveness of gamification in several fields, including education, industry, and healthcare [37]. While this is an emerging research field, several studies have demonstrated the effectiveness of gamified methods as a research strategy. Dopler et al. analysed the use of serious gaming, specifically the differences between digital and in-person strategies [38]. They found that play positively influences cognitive learning outcomes in both online and in-person settings. They did, however, find that face-to-face settings were significantly more effective at improving cognitive learning outcomes, particularly among students in older age groups [39]. This paper looks to explore if gamified methods can have a positive impact on reducing the ‘othering’ of older adults and increase understanding of health and housing inequalities.

There is increasing evidence that serious game methods can support positive outcomes for older adults. Altmeyer et al.’s examination of older adults’ experiences with gamified methods used survey data collected from 18 participants aged 75+, and found that serious games, and gaming overall, could benefit older people [39]. However, they argue that the current predominant design and culture of games, primarily targeting young adults, are significant barriers to their effectiveness. Older people experience gaming in different ways, prioritising socialising, collaborating, and caretaking, over competitive content [39]. The research implies, therefore, that these features should be prioritised when designing motivational gamified methods targeting older adults.

Furthermore, research by McCall et al. examined the potential of serious games to effectively plan for housing and ageing [12]. Employing a ‘serious game’ methodology analysing over 200 policy-maker, practitioner, and service-user perspectives across Scotland, England, and Wales, they uncovered several key issues that must be considered when designing housing for an ageing population. They found that an interdisciplinary approach is vital for producing appropriate housing to cater for older people [12]. Future planning, tackling current needs, and communication across sectors is necessary to centralise housing for ageing, a currently underfunded area, in policy discussions. These would further enable independent living, increased accessibility, and empowerment amongst older people within the housing sector [12]. The researchers found that their serious game methodology was useful in breaking down communication barriers between individuals, and sectors, allowing for a more integrated approach to planning for housing and ageing [12]. Therefore, the use of game-based learning methods certainly represents a useful tool for fostering inclusive, multitiered housing strategies, empowering older adults and professionals in planning for the ageing demographic.

## 2. Materials and Methods

The literature noted that ageism and othering have rarely been examined simultaneously in both an empirical and practical fashion, especially in generating insights around ageing in place. The DesHCA project focused on how we scale up design solutions and implementation to support healthy ageing in place (see [40] for full details on the UKRI/ESRC funded project). As one of the work packages, the DesHCA project co-produced a serious game called ‘Our House’ (described below) to generate discussions about homes and accessibility. The objectives of the research included bringing community groups, older people, practitioners, academics, policy makers and wider stakeholders together in real-world, future-facing experiences to develop a co-produced, exploratory, knowledge exchange tool. This activity prompted key stakeholders to consider the future of housing for themselves and others as we age. As a result, the serious game ‘Our House’ was designed to explore housing for older people which is cognitively sustainable and inclusive. ‘Our House’ is a tool to support data collection and research impact, encouraging people (both individuals and professionals working in the area) to have conversations about housing, ageing, and home design.

This paper presents findings from a sample of 128 participants, with a mixture of housing association (37), academic (13), local government (10), architecture (10), health and care sector (9) charity and third sector (20) and developer (4) backgrounds. There were also older community representatives (25). Participants engaged through a series of 10 playtests throughout the UK, ranging from 5 to 35 people (nine in person and one online).

Participants were recruited namely via housing, health and social care networks and by approaching key organisations that deliver different types of housing (both general needs and housing for older people). To support game delivery, housing associations and tenants’ groups advertised workshop opportunities to multiple stakeholders. Materials for recruitment were diverse and accessible, including videos that showed the game in action to highlight the visual and interactive elements to generate motivation and interest around serious play. There were multiple facilitators from the academic team on hand for support (averaging 4 people per playtest) and every playtest had a community representative in terms of co-production.

The serious game process includes the collection of multiple types of qualitative data, including observation, focus group discussion and semi-structured qualitative questionnaires. Participants are supported in these discussions and decisions by being able to undertake them on behalf of a fictional resident within the game. But they are then also encouraged to use that experience to reflect on their own decisions and planning, and the factors which would be likely to lead to good outcomes.

The serious game was developed co-productively with older adults as partners in the DesHCA project (authors PS, RP, JG). This integrated insights from lived experience through the development and design of the game, alongside representation in 8 of the 10 playtests. Co-production partners brought an important critical challenge to the process, motivated by experiences and insight around local needs, lack of accessibility, and the gap between policy and implementation in local plans that undermined the safety of homes due to the inability to adapt spaces adequately as needs increased. Co-production partners supported analysis and write-up of the findings, with high engagement with the methodology.

The serious game shows that incorporating ageing processes into serious play allows interaction, integration and understanding of how ageing affects people personally. Interaction and discussions facilitated within the serious game enables individuals to project insights around ageing into community and societal settings.

### The Serious Game

**Figure 1:**
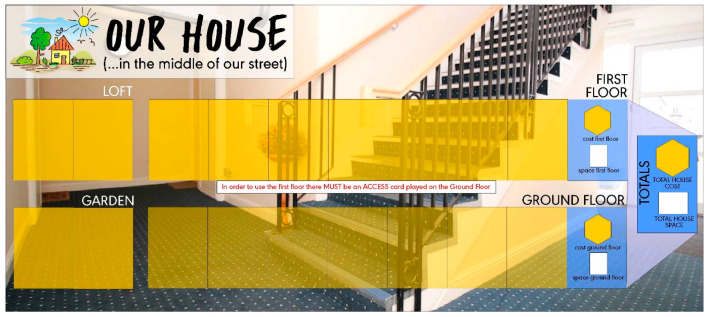
Our House, serious game board visual.

‘Our House’ is played in person, with people in groups of between 6–35. Although it can be played solo, players are usually grouped into pairs to encourage discussion and reflection on the decisions being made. The game provides players with a player mat (Figure 1) representing a house; a deck of cards representing rooms within a house; and a set of adaptation tokens that can be used to adapt houses.

**Figure 2:**
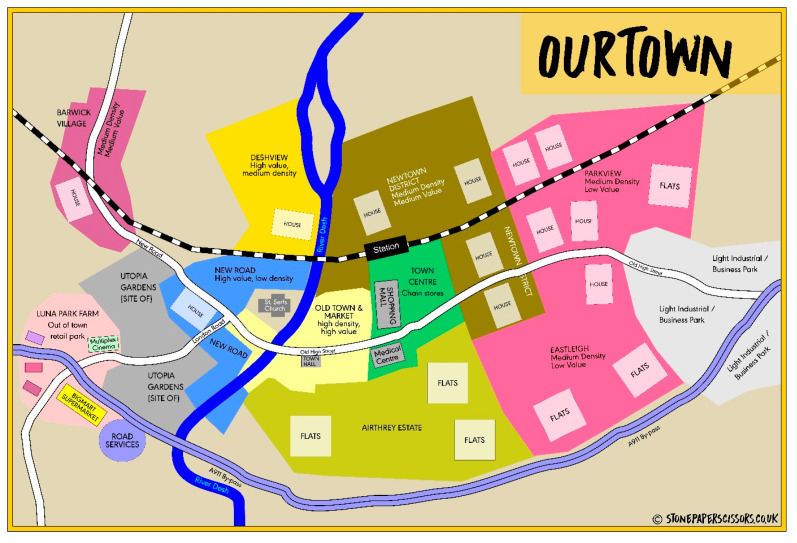
The map of ‘Our Town’.

There is also a central map, which allows all the players to situate their houses within a fictional community (Figure 2).

**Figure 3:**
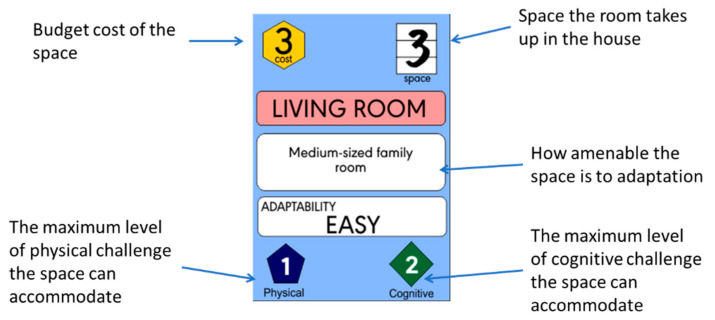
Our House space card.

Each room card is labelled with an evidence-based budget cost, a score for physical accessibility, a score for cognitive accessibility, and an ‘ease of adaptation’ level (Figure 3) potentially providing insight to the longer-term capability of the room to support ageing in place.

In developing this scoring system, the multidisciplinary research team, which included expertise in economics, architectural practice, housing, and dementia-inclusive design, reviewed a wide array of common room attributes, including extended lists of features evidenced to support occupant wellbeing [41,42,43] and triangulated these against their estimated build and adaptation costs [44]. Within this system, larger rooms, with wide doorways, and good lighting levels would tend to score well, may cost more to build, but may be less likely to need adaptations, and be ‘easy’ to adapt where this is required.

The design of the game allows it to be played in multiple ways, but players are encouraged to start with a warm-up exercise of constructing a representation of their own house using the room cards. This helps players to become familiar with the game components whilst prompting them to begin thinking about the accessibility features of their own homes.

**Figure 4:**
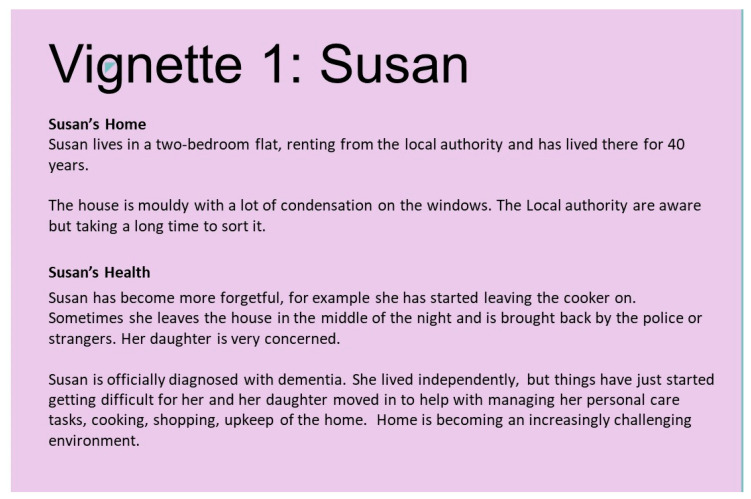
Vignette ‘Susan’ used to play into the future for Our House.

Players are then given a vignette, describing a fictional household (based on real-life examples drawn from the wider DesHCA project). This provides them with a budget, a description of a house, and a description of any needs or challenges that the fictional resident(s) may have (Figure 4). Players use this to construct a suitable house, and to situate it in the community on the central map.

**Figure 5:**
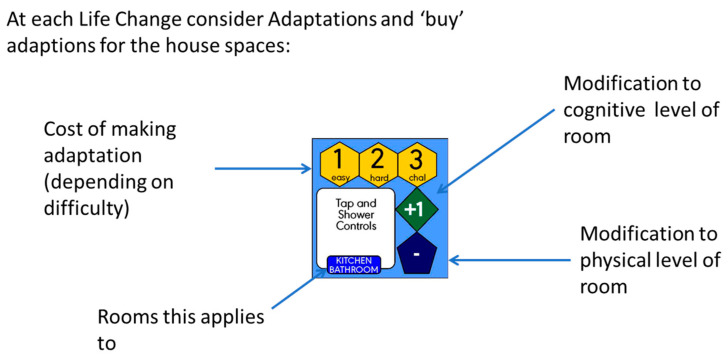
Adaptations tiles that people can purchase in the game Our House to adapt homes and increase accessibility.

In the next turn of the game, players are then given an update on their vignette. This introduces a challenge (perhaps a health condition, accident, or other event) which increases the level of physical and/or cognitive need that a house must support. Players compare the revised need levels of the resident(s) to the accessibility of each room in the house and identify areas of the house that no longer support the needs of their resident(s). Next, an assortment of potential home adaptations is presented, which (for a cost) can increase the physical and/or cognitive accessibility of a room or house (Figure 5).

Players discuss which adaptations might meet the needs of their residents and construct a plan. They must also consider how to fund these: vignettes with wealthier characteristics might have personal access to the resources required, while vignettes with fewer resources must request funding from the local authority (represented by the facilitator). Where adaptations are not possible, do not meet the needs of residents, or are not desirable, players can instead consider moving house. This process is then repeated for a third turn, with a further development in the vignette that increases the challenges facing the residents and requiring that players must respond.

At each stage, players are encouraged to consider and discuss the impact on wellbeing of both the increase in need and the resulting decisions to adapt or move. Following the final turn, a debriefing session is held with the group where players are encouraged to share the story of what happened to their vignette, how they responded, and what these outcomes meant for the resident(s) they were playing. In this debriefing, players also relate what happened to their own homes and situations and reflect on how this might influence choices they make about their own housing.

The serious game method allowed for rich, nuanced, qualitative data to be gathered from facilitation notes, audio recordings of focus group discussions, and feedback forms that were gathered at the end of playtests. The notes, transcripts and feedback forms were analysed firstly by organising feedback into an Excel spreadsheet and transferring qualitative data to QSR NVivo. The data underwent thematic analysis [45] by lead game facilitator (VM) to instigate high-level deductive coding following the wider DesHCA project coding framework linked to key objectives [40]. This was followed by inductive coding of key themes, being open minded and guided by the data starting with facilitator notes, transcripts then feedback forms. Quality checks of the coding and findings were further conducted by note-takers and team members who had been part of the serious game playtests (SJ and MN).

The next section outlines the findings gathered throughout the playtests by exploring the insights gained on the key themes of the othering of ageing, and how serious play can support an increased understanding of healthy cognitive ageing and ageing in place. In the presentation of the findings, we outline data connected to the serious game playtest number (SGPT1, 2 or 3, etc.,) and type of data gathered (notes, focus group, feedback form). Names were not captured due to ethical sensitivities and to allow a safe space to be creative in the game, but respondent numbers are noted in the focus group discussions (R1,2 3, etc., for each discussion). Those who completed feedback forms are given pseudonyms.

## 3. Results

The serious game data showed that engaging people in the ageing process through play allows more interaction, integration and understanding of how ageing affects people personally and enables individuals to project these insights at a community and societal level. Co-productive partners were integrated into the design, delivery, and dissemination of the game, with their feedback focused on the importance of meaningful engagement, and the ability to forge links between key groups such as citizens, planners, higher education, tenants, housing association and more as ‘*opportunities for time together across silos is gold-dust*’ (quote from co-production partner). The incorporation of play with ageing makes the process and concept more tangible, resulting in serious play with a concept (rather than an environment) and helping to overcome the othering of ageing across stakeholders.

### 3.1. The ‘Othering’ of Ageing

Professionals, older people and wider stakeholders tend to connect to ageing in a process of ‘othering’. Data from the serious gameplay offered a microcosm of the thought processes in terms of how people understand and respond to ageing. The findings outlined many different perspectives, illustrating professionals who negotiated situations and scenarios to entice reactions and discussions on a personal and professional level. For example, professionals often felt removed, or approached the vignettes in a professional capacity rather than projecting circumstances onto themselves (SGPT1, Notes).

The gaming scenario highlighted how easy it was to step back from the personal experience of ageing. Participants would often utilise deflective mechanisms and only focus on their professional perspectives rather than discuss that they themselves are ageing. This othering of ageing in the game was consistently highlighted as something practitioners experience in practice, which made future proofing for housing more difficult for practitioners.

*But people don’t want to think about it. Especially when you start offending people and saying that an older person is aged 55 and older, and it’s like, but I’m not old.*

(R10, SGPT5, focus group)

*I suppose if you start talking about ageing, at a younger age, it’s less sort of that whole kind of elephant in the room, people don’t want to talk about it. If it’s part of design, and it’s made to look nicer, then people aren’t going to be so, like, oh I’m not making my house an old person’s house.*

(R12, SGPT2, focus group)

The ’othering’ of ageing was linked to the intangibility of the concept itself, and the multidimensionality of the solutions that can and need to be developed to support an ageing population and people to age well at home:

*I guess, maybe how you can see with age, it’s overwhelming, as well, like it’s new, adapting your house, and all the different options that will be there for you, it’s probably quite a lot for somebody to get into, and then funding streams, and things.*

(R6, SGPT5, focus group)

The othering of ageing has an interesting relationship with budgetary and financial discussions around cost. Talking about finance of adaptations ‘de-personalised’ the story, narratives, impact, and outcomes related to the home and adaptations. Notes from playtest 1, for example, notes that participants were practically and financially orientated with less focus on the personal side of the making the home suitable, focusing on costs that fit the characters’ budget (SGPT1, Notes).

Focusing on funding and finance made it easier to plan but did have an impact on more person-centred approaches to ageing. Participants acknowledged consistently the importance of finance, cost, and budgets but this could lead to two-tier processes and inequality.

*I think the main thing for us was, see just the budget, I think it kind of highlights just how important it is to have access to money… it sums up just how kind of a two-tier society you’ve got.*

(R6, SGPT2, focus group)

To summarise, the ‘othering’ of ageing was an interesting aspect of the professionalisation surrounding services for ageing. Participants led by budgetary considerations often overrode personalising the process and thinking about the individual. There was consideration of how difficult people find it to personalise ageing, and this was a key barrier faced by those trying to deliver housing, care and health services, especially in areas of futureproofing for ageing.

### 3.2. Personalising Ageing by Serious Gaming

The previous section highlighted that many professionals who develop services to support the ageing demographic often do not personalise the ageing process, nor attach it to themselves, and also work with people to ‘other’ the process of ageing. This can be a key barrier to planning.

The serious game personalised this process in several ways. Firstly, through connecting to real-world-based vignettes; secondly, applying vignette circumstances to personal and family connections and professional roles; and thirdly, starting to think about their own homes and future. This section offers the evidence relating to these three mechanisms for the personalisation of ageing.

The gaming scenario encourages people to live through fictionalised vignettes based on real people and evidence. Through the game, people become very connected to these individuals, their stories and invested in their life changes. For example, from playtest 5, “*we had lots of angst, because we didn’t, we knew Ben [in the vignette] wouldn’t really want to move*” (SGPT4, Notes). Individuals could then apply these scenarios to other networks around them, often applying them to changes in their personal lives or networks:

*It makes me think about my parents, now, and thinking, okay, their house is not currently fit for ageing well…. And that’s scary.*

(R5, SGPT6 focus group)

*Well, am I going to have white, plastic grab-rails everywhere, is my home going to become ugly… I feel like it’s a hard sell, to people over the age of 55, 60, you know. I’m thinking of my own mum, she’s like, well I’m not an old person, you know, she’s only mid-60s.*

(R10, SGPT5, focus group)

Secondly, the game offers a safe and creative space to think through personal experiences of ageing. However, even the gaming scenario brings up sensitivities:

*I think I’ve just built Derek’s [in the vignette] whole life up. And I just find it, it’s just a really hard thing to sit and talk about, abstractedly, when you have experienced this yourself in your own family, and it isn’t a paper exercise, that’s quite hard.*

(R2, SGPT6, focus group)

*Because life does happen, it does change. I speak from experience. My partner was diagnosed with MND, which I mean, that just turns your world on its head… And well, adaptations, that doesn’t even begin to cover what was needed.*

(R3, SGPT9, focus group)

Thirdly, the game encouraged people to think about their own homes, but this was a rarer occurrence. Participants struggled when the game mechanisms asked them to think through how they would plan for themselves, rather than others. For example, participants “*said they would feel frustrated*” when asked if they would be happy if this was their home (SGPT2, Notes).

This point is well made in the following feedback from a participant who was making this point themselves but still deploying the language and positioning of ‘the other’:

*All of a sudden you… start bandying around that people are old, you know, you’re an old person, you need to consider old people things, and it’s like, no, no I don’t, I’m perfectly fine… but it’s not a negative thing, it’s a positive thing, and it’s trying to make sure that we don’t get to, kind of, crisis point.*

(R11, SGPT5, focus group)

*Everybody thinks of somebody who’s old is ten years older than them… So, I’ve just turned 50, so I don’t see myself as old, but you know, 60, and I still won’t see myself as old.*

(R1, SGPT5, focus group)

This reinforces the power of ‘othering’ as it was still much more common, and easier, for participants to think of ‘the other’ rather than apply the learning to their own future situations. This links to earlier insights around the consequences of ageism and the processes of self-ageism/self-othering and thinking about the future in older age.

### 3.3. Serious Play and Ageing

The previous sections have highlighted the strength of the process of othering, and how the serious game mechanism allows for personalisation at different levels and encourages an ageing-in-place perspective. This was highlighted particularly in the consideration that participants took in language and feedback around future-proofing homes and environments.

*Today’s visit brought home the importance of future proofing.*

(Aaron, SGPT1, feedback form)

The game mechanisms further allowed for a more in-depth and nuanced consideration of ageing and perspectives around ageing:

*But that’s all wrapped up with how people conceive ageing, isn’t it? And I think we need to change the conversation, so that people see ageing in a positive way, you know, the joke being, it’s better than the alternative. But, you know, it is about changing that, the whole conversation around ageing.*

(R11, SGPT5, focus group)

Importantly, the game board scenario of looking at individuals’ homes over time encouraged a shift in what the home is for—more in line with being a ‘home’ over simply a ’house’:

*The exercise has made people to think about house as a place to get the most out of rather than as an asset to be passed on.*

(SGPT5, notes)

*Does that still feel like a home, or is it a place that you’re surviving in.*

(R1, SGPT6, focus group)

At its core, the serious game methodology is a knowledge exchange tool. The game mechanisms are there to encourage creative discussion, connection, and networking. Playtests in locations where there were multiple stakeholders across different organisations, or organisational departments, were often the most effective. In discussion points, participants were often learning about what each other did, especially across key areas such as health, housing, and social care. Through the game and narratives, there was positive feedback in people learning about differences between tenure, funding streams, and adaptations:

*I think it highlighted for me… working from the social housing side of things, how much easier it is for a tenant of a social landlord to get an adaptation. But actually, conversely, how difficult it is for someone living in private accommodation.*

(SGPT5, focus group)

*This has prompted me to think more thoroughly about the ways a home can adversely impact an individual’s wellbeing… I will aim to promote this future-oriented perspective as I continue into my profession. I recommend continued collaboration between OT [occupational therapist] + housing officers in order to promote holistic wellbeing for the clients.*

(Erika, SGPT1, feedback form)

There was also evidence of impact on professional perspectives, where the storification and game narrative allowed a paradigm shift in thinking about what is best for supporting individuals’ cognitive and physical changes. In one example, participants looking after the vignette ‘Susan’ (Figure 4) from a policing background, were very concerned around safety and were negotiating the safety elements of house location, adaptations, and technology. They noted that if looking at this from their police officer perspective, usual practice would mean that Susan would have to move for the safety of her and others. However, the serious game challenged their perspective on the solution for support when taking it from a personal perspective:

*So, from a policing perspective, what would happen in Susan’s case… we would notify social services, and the housing, the social housing she was actually in, and notify them of the actual incident. And that will probably trigger conversations from a multiagency partnership approach to whether Susan’s accommodation is suitable for her, or if she was placing herself more at risk. Which, looking at it from this perspective now, and thinking of me being Susan, it’s totally against what I’ve been dealing with for the last 27 years.*

(R1, SGPT5, focus group)

When taking on the persona of the individual ‘Susan’, they concluded that they would decide to act contrary to 27 years of their professional experience and aim to ‘age in place’ in their current home. In the feedback forms, participants were asked how the experience of the Serious game would then be taken away in their professional and personal lives. Interestingly, many confirmed they would go and review their own processes and practice:

*Make allowance for future proofing before health starts to decline. Think about space standards and ease of adaptability. Look at own design guide to make more/all homes adaptive.*

(Henry, SGPT1 feedback from)

Much of the feedback for future planning around professional practice was linked to the elements explored in the game, for example, issues of delays waiting for diagnosis:

*Find small aids that help me and complement my supported housing. Develop support package while waiting for a diagnosis. Create + develop (new) shared places, e.g., shared gardens.*

(Nelly, SGPT2, feedback form)

*Thinking towards future house moves for various physical and cognitive problems. b: personalising projects and future end users. Using capital costs to reduce operational/maintenance costs. Embedding adaptability as a requirement at briefing stage.*

(Pierre, SGPT2, feedback form)

The serious game was seen as a mechanism that helped overcome othering, internalize and understand various lenses around the concept of healthy ageing:

*We do fixate about age, but we don’t focus as much about people’s health. And actually, things can happen to any of us, at any age… that suddenly means that we’ve got all of these physical barriers in our home. And I presume that we probably all know people that something has happened to them, whether they’ve had a stroke, or you know, and then overnight, the home is just not accessible. So, it’s maybe just taking that age, you know, assuming it’s all tied in with ageing, and actually, it’s just about being able to live healthy and independently, and to have a home that allows you to do that. And maybe that’s where the discussion needs to be, you know, around that. And it could be anybody in your household, it could be a child, it could be, you know, yourself.*

(R3, SGPT5, focus group)

Overall, the serious game allowed key paradigm shifts in the direction of tangible, actionable ideas around the conceptualisation of ageing in housing, health, and social care practice. The process of playing into the future helped with the internalisation of ageing and joining the dots between personal and professional experience.

### 3.4. The Challenges for Planning for the Future

The serious game allows for a discussion of real-life scenarios, mirroring barriers and challenges in real life provision around home, health, social care, and ageing. However, even after giving the creative space to explore, limitations were clear in the negotiations within the game. Throughout the playtests it was noted that:

*And that is, you know, I guess, ideal solutions do exist, but in reality, they don’t, because people will always have some kind of constraint. Whether that’s a budgetary constraint on their own finances, or whether their grant applications don’t get accepted, or the type of property that they’re in isn’t conducive to adaptations, there’s always going to be some kind of compromise.*

(R10, SGPT5, focus group)

There were numerous structural forces discussed that contributed to inertia in planning for the future and future-proofing homes. In particular, not having an official diagnosis for health support needs was seen as a key barrier:

*Well, I think it’s tricky with him, because he doesn’t have a diagnosis, so we’re not a hundred percent sure. At least with a diagnosis, you might have a bit of a clearer idea about what, if there is going to be further deterioration, what that means for your body, and what, then, future-proofing you might need.*

(R1, SGPT6, focus group)

In conclusion, “*there’s always going to be compromise*” (SGPT5, notes) and the serious games allows that exploration of priorities and negotiation. Feedback from the co-productive partners focused on the importance and impact of meaningful engagement where “*serious games are a much-needed link and spark thoughts, as well as forge networks*”. However, although gaming scenarios can help discover and think through the challenges, the solutions are restrained in that the wider picture in overcoming the consequences of ageism is a societal challenge with multilayered solutions. The ‘othering’ of ageing has been shown in this paper to be one of the key barriers and overcoming the ‘othering’ of ageing and taking personalised approaches are key to supporting change within policy and practice.

## 4. Discussion

The findings from the serious game playtests highlight that housing professionals and wider stakeholders engage consistently in the ‘othering’ of ageing. There were clear processes of self-ageism/self-othering, where professionals and older people found it difficult to apply the process of ageing to themselves. The consequences of this include a certain ‘abjection’ in planning for ageing and exploring solutions, as the challenge is seen as too wide, too intangible, and framed as something that can never be overcome.

The co-productive nature of the project and the methodological development emphasised that the key to overcoming barriers is about “joining the dots” (quote from co-production partner) across sectors and between citizens. The serious game shows that further enabling of independent living is linked to adaptations for both cognitive and physical impairment, which need to be considered when adapting to changing or special needs. Furthermore, considering the implications of the COVID-19 pandemic that arrived in the UK in 2020, the need for partnership, interprofessional and multiagency working is even more of a priority for housing delivery. Organisations across housing, health and social care need to work together to deliver and plan for the ageing population effectively.

The serious game method used in the project showed how the ‘othering’ of older people reflected in what people shared about housing, health and social care practice. Participants negotiated the link between both their future selves and the futures of others. For example, often people were talking about a home or adaptation process being good for a different group—such as someone with dementia, or a wheelchair user. This links to the consistent framing of sub-groups of older people in ways that relate negatively to ageism [1,17]. This is clear as when participants are further asked about living in such an environment themselves, they would not agree to it being their own home—or would feel ‘frustrated’ living there. Furthermore, although the game offered a creative space to think through solutions to ageing and support, participants consistently applied barriers such as budgets and finance of adaptations to ‘de-personalise’ the story, narratives, impact, and outcomes related to the home. This translated to barriers in thinking about the future in older age as ‘othering’ that can result in fewer person-centred housing decisions without consideration of the nuanced solutions to ‘ageing in place’.

The process of playing the serious game highlighted several ways in which the game also challenges this ‘de-personalisation’ of ageing. For example, where a participant took the persona of the individual ‘Susan’, they concluded that they would decide to act contrary to 27 years of their professional experience and aim to ‘age in place’ in their current home. There was also a lot of connection between vignette situations, personal experiences, and experiences with parents who are growing older. This supports the insights in the literature around the serious game method being successful at solidifying ideas, encouraging creative thinking, improving cognitive learning outcomes, and helping think through integrated approaches to housing and ageing [12,34,38]. In this way, looking to the future and the idea of ‘belonging’ in the future [46]—not associated with ideas of ‘decline’ [29]—is an important way to connect self-agency to key elements of healthy ageing.

There are of course limitations to such an approach, as gameplay highlights that there are numerous structural forces discussed that contributed to a lack of planning for the future and future-proofing homes. These were often very practical, for example, not having an official diagnosis for health support needs. Also, at times, a gaming approach results in people focusing more on the mechanisms, rather than the discussion and paradigm shift looking to be initiated. Furthermore, the real-world scenarios can at times cross over to being too personal or apply a little too closely to challenges people are facing in real life that they find upsetting. This does reinforce, however, the importance of understanding the process of ageing.

Implications for policy and practice from the findings include, firstly, that ageism and ‘othering’ are prevalent in current approaches to delivering housing, health and social care services and hinder planning for the future. The paper shows that co-production approaches are key in developing effective interventions, solutions and insight. The findings indicate that tangible opportunities to play into the future via real-world scenarios support the paradigm shift needed to combat larger challenges around healthy ageing. There is a need for more space in both policy and practice development that allows for strategic and creative approaches to plan for ageing. The paper shows the potential for practical tools such as serious games to bridge the gap between policy and practice around ageing well in place.

## 5. Conclusions

The findings show positive impacts in relation to the question around the ability of creative methodologies such as serious gaming in challenging the ‘othering’ of ageing on a professional and personal level. The serious game personalised and enabled connection to real-world based evidence via vignettes and playing through future transitions. Applying various circumstances and evidence supported people to think about their own homes and future.

The paper concludes that the serious gaming of ageing encourages people to think differently about the concept of healthy ageing—both physically and cognitively. This then allows us to consider scalable/creative solutions to prepare for ageing that bridge the gap between the personal and the private. Overcoming the ‘othering’ of ageing is important for finding tangible solutions that support everyone as health dynamics change, and as we all—inevitably—grow older. Challenging ‘othering’ also counters the negative consequences of ageism, overcoming stereotyping and trying to help people overcome restrictions—both real and perceived—on access to resources that support healthy ageing.

In this particular approach, the serious play supports increased understanding of healthy cognitive ageing and ageing in place. It makes the process and concept of healthy ageing more tangible, overcoming the ‘othering’ of ageing. Overall, the serious game was seen as a mechanism that helped to internalize and understand various perspectives around the concept of healthy ageing.

## Data Availability

The data that support the findings of this study are openly available in DataSTORRE, the Stirling Online Repository for Research Data, University of Stirling. Full reference for the Dataset: McCall, V; Rutherford, A. Serious game data archive for the Designing for Healthy Cognitive Ageing (DesHCA) Project. Version 1. University of Stirling, Faculty of Social Sciences. 2024. Dataset. http://hdl.handle.net/11667/227 (accessed on 1 March 2024).
